# Emergence of heterogeneity in acute leukemias

**DOI:** 10.1186/s13062-016-0154-1

**Published:** 2016-10-12

**Authors:** Thomas Stiehl, Christoph Lutz, Anna Marciniak-Czochra

**Affiliations:** 1 Institute of Applied Mathematics, Heidelberg University, Im Neuenheimer Feld 205, Heidelberg, 69120 Germany; 2 Interdisciplinary Center for Scientific Computing, Heidelberg University, Im Neuenheimer Feld 205, Heidelberg, 69120 Germany; 3 Bioquant Center, Heidelberg University, Im Neuenheimer Feld 297, Heidelberg, 69120 Germany; 4 Department of Medicine V, Heidelberg University, Im Neuenheimer Feld 410, Heidelberg, 69120 Germany

**Keywords:** Acute leukemia, Heterogeneity, Clonal evolution, Mathematical modeling, Patient prognosis, Mutation, Self-renewal, Clonal hierarchy

## Abstract

**Background:**

Leukemias are malignant proliferative disorders of the blood forming system. Sequencing studies demonstrate that the leukemic cell population consists of multiple clones. The genetic relationship between the different clones, referred to as the clonal hierarchy, shows high interindividual variability. So far, the source of this heterogeneity and its clinical relevance remain unknown. We propose a mathematical model to study the emergence and evolution of clonal heterogeneity in acute leukemias. The model allows linking properties of leukemic clones in terms of self-renewal and proliferation rates to the structure of the clonal hierarchy.

**Results:**

Computer simulations imply that the self-renewal potential of the first emerging leukemic clone has a major impact on the total number of leukemic clones and on the structure of their hierarchy. With increasing depth of the clonal hierarchy the self-renewal of leukemic clones increases, whereas the proliferation rates do not change significantly. The emergence of deep clonal hierarchies is a complex process that is facilitated by a cooperativity of different mutations.

**Conclusion:**

Comparison of patient data and simulation results suggests that the self-renewal of leukemic clones increases with the emergence of clonal heterogeneity. The structure of the clonal hierarchy may serve as a marker for patient prognosis.

**Reviewers:**

This article was reviewed by Marek Kimmel, Tommaso Lorenzi and Tomasz Lipniacki.

## Background

Acute leukemias are clonal diseases of the blood forming (hematopoietic) system. They lead to expansion of malignant cells and resulting impairment of blood cell formation. Over the last years evidence has accumulated that many leukemia subtypes are maintained by a subpopulation of cells with stem cell-like properties [1–3]. These cells are referred to as leukemic stem cells (LSCs) or leukemia initiating cells (LICs) and potentially trigger relapse of the disease [4, 5]. Recent sequencing studies have confirmed that the leukemic cell population is composed of different clones [6–8]. The size and number of clones follows a complex evolution over the course of the disease [9–12]. Genetic heterogeneity of different clones seems to result in functional differences, such as a different engraftment potential in mice or different proliferation rates [13, 14]. Nevertheless, a direct link between genotype and cell function is still missing [13].

**Fig. 1:**
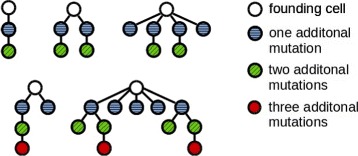
Examples of the clonal architecture detected in leukemic patients. Each tree corresponds to one patient. The cell at the top corresponds to the leukemic founder cell which acquires additional mutations and gives rise to multiple leukemic clones. The examples are taken from [12]. Reconstruction of the clonal architecture from genetic measurements is not always unique. In case of ambiguity only one possibility is shown in the figure

Genetic instability is a hallmark of solid cancers but a relatively rare event in acute leukemias. The number of somatic mutations detected in acute leukemias is small compared to most other cancers [15, 16]. Nevertheless acute leukemias show a considerable interindividual genetic heterogeneity and a complex genetic relationship among the different clones. The clonal architecture of leukemias shows high interindividual variability [12], see Fig. 1 for examples. The source of this variability is so far unknown.

Clonal evolution in leukemias is a complex process. Hematopoiesis is known to be a tightly regulated process subject to several nonlinear feedback mechanisms [17]. Leukemic cells of many patients have the potential to interact with hematopoietic feedback signals [18, 19]. This may include leukemic cell stimulation by hematopoietic factors as well as alteration of the concentration of feedback signals by leukemic cells. Experiments further suggest the interaction of leukemic cells with the bone marrow microenvironment [20, 21]. Nonlinear interactions between hematopoiesis and the leukemic cell expansion on one hand and the limited bone marrow space on the other may influence clonal selection [22]. The fact that important cell parameters such as proliferation rates or self-renewal probability cannot be measured in vivo further limits our understanding of leukemia evolution. Especially the following questions are so far unresolved:

- What is the source of interindividual differences of the clonal hierarchy of leukemias?
- What is the functional difference in terms of self-renewal and proliferation rates between cells at the top of the hierarchy and their descendants which have acquired additional mutations?
- How do clones that appear early during the disease differ from clones that appear later?
- How do properties of leukemic cells present at one point in time influence the structure of the clonal hierarchy in the future?
- How do mutation rates influence the structure of the clonal hierarchy?

These questions are of clinical relevance, since properties of the leukemic stem cells are important determinants of disease dynamics, therapy resistance and relapse [14, 22, 23]. Deciphering of the clonal architecture using genomic methods has become more efficient and less expensive in recent years. Prediction of patient prognosis based on genetic markers alone is not straightforward, since leukemogenetic hits vary considerably among patients and the interplay of the different detected mutations is complex and only partially understood [24, 25]. Therefore, the question arises if the structure of the clonal architecture provides additional insights into cell properties and patient prognosis.

We propose a mathematical modeling approach to provide potential answers to these questions. Mathematical models allow to systematically study the impact of cell parameters such as mutation rates, proliferation rates and self-renewal probability on the clonal hierarchy of cells. Simulation of the clonal selection process provides hints about which cell properties are linked to selective advantage and how these properties evolve over time. The modeling approach allows linking the position of a clone within the hierarchy to functional properties, such as self-renewal and proliferation rates, and to compare it to functional properties of clones located at different positions in the hierarchy. The structure of the clonal architecture obtained in model simulations is compared to experimental data from the literature and thus allows linking observations at the level of population dynamics to the cell function in terms of self-renewal and proliferation rates.

Different mathematical concepts have been proposed to model mutations. Depending on the focus of interest, different approaches can be applied. Moran process [26, 27] is used to describe populations with size that is constant in time. Branching processes are used to describe acquisition of mutations in growing populations. Examples include the accumulation of passenger and driver mutations, interaction among driver mutations or accumulation of mutations during DNA copying [28–31]. In case of a large population and a continuous trait space, diffusion [32, 33] or integral kernels [34–36] have been used to describe the effect of mutations. A rigorous relationship between processes at the level of single cells and limit descriptions in terms of deterministic or probabilistic equations is provided in ref. [37]. Examples for deterministic approaches to study mutations in discrete or continuously structured population models are given in ref. [38–40]. Computer simulations of individual-based models and cellular automata provide a framework to study the impact of cellular processes on the whole population. Examples for individual-based cancer models can be found in ref. [41, 42].

This work is structured as follows. In the “Methods” section, we introduce the mathematical model. In the “Results” section, we present simulation results and their biological interpretation. The “Discussion” section concludes with a discussion of clinical implications of the results obtained.

We adhere to the following terminology. Clonal architecture (clonal hierarchy) is understood as the genetic relationship between different clones. We represent the clonal architecture as a tree. Progeny of a node has acquired one additional mutation compared to its mother node. As a clone we understand all genetically identical stem and non-stem cells. A clone consisting of at least 1 % of the total cell mass is denoted as a significant clone. The threshold of 1 % has been chosen based on the sensitivity of sequencing methods [43].

## Methods

**Fig. 2:**
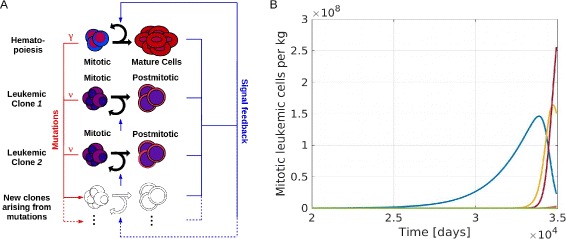
Overview of the mathematical model. **a** Model structure: The model includes one hematopoietic cell line and an arbitrary number of leukemic clones. Leukemic and healthy cells interact by feedback signals. Due to mutations new clones with different properties arise. Mutation rates of leukemic and healthy cells are denoted as *ν* and *γ* resp. **b** Example simulation: The panel shows the time course of mitotic leukemic cells. The horizontal axis shows the time since appearance of the first leukemic cell. The simulation ends when the mature healthy cell count is below 5 *%* of its steady state value. This corresponds to the death of the patient. Each color represents one clone

The model is defined as a system of nonlinear ordinary differential equations describing time evolution of hematopoietic cells and leukemic clones. Experimental data imply that hematopoietic and leukemic cells interact, e.g., through feedback signals or the bone marrow microenvironment [18–21]. Therefore, the model takes into account both healthy and leukemic cells. The presented model is an extension of the models of healthy hematopoiesis [44–46] and acute leukemias [22, 23, 47]. The main novelty lies in considering a time dependent number of leukemic clones and in tracking the structure of the clonal hierarchies. During the course of the disease new clones arise due to mutations which are acquired by leukemic cells. Properties of new clones are chosen from random distributions that depend on the properties of the cells that give rise to them. To model stochastic extinction of clones with favorable properties, we take into account their extinction probabilities using the theory of branching processes. Compared to the work presented in [40], which focuses on neutral mutations in non-stem cells without feedback regulation or competition, we are interested in the evolution of non-neutral stem cell mutations under competitive pressure of a nonlinear feedback mechanism. An overview of the model is provided in Fig. 2 a.

### Model structure

Based on the classical understanding of the hematopoietic system [48] blood cell formation is considered as a stepwise process, with cells sequentially traversing an ordered sequence of discrete maturation states (compartments). We treat each compartment as a “well-mixed tank” and describe its evolution using an ordinary differential equation. The large count of cells in the hematopoietic system justifies this approach [48].

Since most leukemias are diseases of the white blood cells, we only consider the white cell lineage of the healthy hematopoietic system. The model describes the interaction of the healthy cell lineage with an arbitrary number of leukemic clones. We assume that each lineage or clone consists of two different cell types, namely cells that are able to divide (stem and progenitor cells) and cells that have lost the ability to divide (mature cells or post-mitotic leukemic blasts). Each cell type is characterized by the following cell properties:

- Proliferation rate, describing the frequency of cell divisions per unit of time. In case of post-mitotic cells the proliferation rate is considered equal to zero.
- Fraction of self-renewal (self-renewal rate), describing the fraction of progeny cells returning to the compartment occupied by the parent cells that gave rise to them.
- Death rate, describing the fraction of cells dying per unit of time. For simplicity, we assume that dividing cells do not die and that non-dividing cells die at constant rates.

We denote the compartment of dividing healthy cells as *c*_1_ and that of mature cells as *c*_2_. We count the leukemic clones starting from 1. The respective compartments of the *ith* leukemic clone are denoted as ${l_{1}^{i}}$ and ${l_{2}^{i}}$ resp. The proliferation rate of the healthy cells is denoted as *p*_*c*_ and that of the mitotic cells of the *ith* leukemic clone ${p^{i}_{l}}$. The respective fractions of self-renewal are denoted *a*_*c*_ and ${a_{l}^{i}}$. Death rates of the non-dividing compartments are *d*_*c*_ and ${d^{l}_{i}}$.

### Feedback regulation of healthy hematopoiesis

Formation of healthy blood cells is subject to a tight regulation, mediated by a system of lineage- and stage-specific cytokines. If there is a need for more blood cells of a certain type, the concentration of cytokines increases and stimulates formation of mature cells [17, 49]. For simplicity, we consider only one feedback loop. We denote *s*(*t*) the value of the feedback signal at time *t*. We set $s(t)=\frac {1}{1+{kc}_{2}(t)}$, where *k* is a positive constant depending on production and elimination of cytokines [44]. This expression can be derived from cytokine kinetics [44]. It takes into account that the concentrations of important cytokines such as EPO and G-CSF depend on the concentration of mature cells [49]. The feedback signal assumes values between 0 and 1.

On the basis of our earlier work and compatibility with clinical data [44, 46], we assume feedback inhibition of the fraction of self-renewal by mature cells. The fraction of self-renewal of the healthy cells is assumed to be equal to $a_{c}(t)=\hat a_{c} s(t)$ and that of leukemic cells of clone *i* to ${a_{l}^{i}}(t)=\hat {a}_{l}^{i} s(t)$. The parameters $\hat {a}_{c}$ and $\hat {a}_{l}^{i}$ can be interpreted as the maximal possible fraction of self-renewal. Numerical solutions of the model of hematopoiesis subject to this feedback were validated on the basis of clinical observations and show good agreement with patient data upon recovery from bone marrow transplantation [46].

### Model equations for the hematopoietic system

The flux to mitosis of healthy cells at time *t* equals *p*_*c*_ *c*_1_(*t*). During mitosis, a parent cell is replaced by two progeny cells. The outflux from mitosis at time *t* equals 2*p*_*c*_ *c*_1_(*t*), of which the fraction $2\hat {a}_{c} s(t){p_{c}}c_{1}(t)$ stays in compartment 1 (process referred to as self-renewal). The fraction $2\left (1-\hat {a}_{c} s(t)\right){p_{c}}c_{1}(t)$ moves to compartment 2 (process referred to as differentiation).

We obtain the following system of ordinary differential equations

$$\begin{array}{@{}rcl@{}} \frac{d}{dt}c_{1}(t)&=&\left(2\hat{a}_{c}s(t)-1\right)p_{c}c_{1}(t)\\ \frac{d}{dt}c_{2}(t)&=&2\left(1-\hat{a}_{c}s(t)\right)p_{c}c_{1}(t)-d_{c}c_{2}(t)\\ s(t)&=&\frac{1}{1+{kc}_{2}(t)} \end{array} $$

with the initial conditions *c*_1_(0), *c*_2_(0) given.

### Model of leukemia

We assume that healthy and leukemic cells respond to the same feedback signals. This assumption is supported by the finding that leukemic cells express receptors for hematopoietic cytokines [18] and that they interact with the bone marrow microenvironment [20, 21]. Due to cytokine clearance by receptor mediated endocytosis [17, 49] leukemic cells contribute to the cytokine clearance. In the presence of leukemic cells, the feedback signal is given by

$$s(t)=\frac{1}{1+{kc}_{2}+ k\sum_{i=1}^{n(t)} {l_{2}^{i}}(t)}. $$

Here, *n*(*t*) denotes the number of leukemic clones present at time *t*. This expression has been derived in ref. [47] for the special case of one leukemic clone. The proposed feedback mechanism has been validated based on clinical data [23]. Model simulations suggest that the choice of individual *k* values for each leukemic clones, i.e., modeling the signal as $s(t)=\frac {1}{1+{kc}_{2}+ \sum _{i=1}^{n(t)} k^{i} {l_{2}^{i}}(t)} $ has no significant impact on the quantities considered in this study. For *n* leukemic clones we obtain the following system of equations:

$$\begin{array}{@{}rcl@{}} \frac{d}{dt}c_{1}(t)&=&\left(2\hat{a}_{c}s(t)-1\right)p_{c}c_{1}(t)\\ \frac{d}{dt}c_{2}(t)&=&2\left(1-\hat{a}_{c}s(t)\right)p_{c}c_{1}(t)-d_{c}c_{2}(t)\\ \frac{d}{dt}{l^{1}_{1}}(t)&=&\left(2\hat{a}_{l}^{1}s(t)-1\right){p_{l}^{1}}{l^{1}_{1}}(t)\\ \frac{d}{dt}{l^{1}_{2}}(t)&=&2\left(1-\hat{a}_{l}^{1}s(t)\right){p_{l}^{1}}{l^{1}_{1}}(t)-{d_{l}^{1}}{l^{1}_{2}}(t)\\ \vdots&\vdots&\vdots\\ \frac{d}{dt}{l^{n}_{1}}(t)&=&\left(2\hat{a}_{l}^{n}s(t)-1\right){p_{l}^{n}}{l^{n}_{1}}(t)\\ \frac{d}{dt}{l^{n}_{2}}(t)&=&2\left(1-\hat{a}_{l}^{n}s(t)\right){p_{l}^{n}}{l^{n}_{1}}(t)-{d_{l}^{n}}{l^{n}_{2}}(t)\\ s(t)&=&\frac{1}{1+{kc}_{2}(t)+k\sum_{i=1}^{n} {l_{2}^{i}}} \end{array} $$

with the initial conditions *c*_1_(0), …, ${l_{2}^{n}}(0)$ given.

### Mutations

We assume that mutations occur during genome replication which takes place before mitosis. We consider the rate to be identical for all clones and constant in time. This is supported by the fact that genomic instability is a relatively rare event in leukemias [15, 16]. The flux to mitosis of leukemic clone *i* at time *t* is given as ${p_{l}^{i}}{l_{1}^{i}}(t)$. We assume that a fraction *ν* of the produced progeny has a mutation. Therefore, $2{p_{l}^{i}}{l_{1}^{i}}(t)\nu $ mutated cells are produced at time *t*, of which $2{{\hat {a}_{l}^{i}}}s(t){p_{l}^{i}}{l_{1}^{i}}(t)\nu $ are in the mitotic compartment and $2(1-{\hat {a}_{l}^{i}}s(t)){p_{l}^{i}}{l_{1}^{i}}(t)\nu $ belong to the post-mitotic compartment. The influx of mutated mitotic cells from the clone *i* is defined as $\alpha _{i}(t)=2{\hat {a}_{l}^{i}}s(t){p_{l}^{i}}{l_{1}^{i}}(t)\nu $. The number of non-mutated cells is given by $2{p_{l}^{i}}{l_{1}^{i}}(t)(1-\nu)$, of which $2{\hat {a}_{l}^{i}}s(t){p_{l}^{i}}{l_{1}^{i}}(t)(1-\nu)$ are mitotic cells and the remainder, $2(1-{\hat {a}_{l}^{i}}s(t)){p_{l}^{i}}{l_{1}^{i}}(t)(1-\nu)$, belongs to the non-dividing compartment. We obtain the following set of equations describing dynamics of clone *i*:

$$\begin{array}{@{}rcl@{}} \frac{d}{dt} {l_{1}^{i}}(t)&=2{\hat{a}_{l}^{i}}s(t){p_{l}^{i}}{l_{1}^{i}}(t)(1-\nu) -{p_{l}^{i}}{l^{i}_{1}}(t)\\ \frac{d}{dt}{l_{2}^{i}}(t)&=2(1-{\hat{a}_{l}^{i}}s(t)){p_{l}^{i}}{l_{1}^{i}}(t)-{d_{l}^{i}}{l_{2}^{i}}(t)\\ \alpha_{i}(t)&=2{\hat{a}_{l}^{i}}s(t){p_{l}^{i}}{l_{1}^{i}}(t)\nu \end{array} $$

A similar system of equations has been obtained in [40].

Since ${l^{i}_{2}}$ is considered to be post-mitotic, we do not distinguish between cells that acquired a mutation during the divisions and those that did not. The influx *α*(*t*) of mutated mitotic cells of all leukemic clones at time *t* is given by $\alpha (t)=\sum _{i=1}^{n(t)} \alpha _{i}(t)$, where *n*(*t*) is the number of leukemic clones present at time *t*.

We consider the rate *α*(*t*) as the rate of an inhomogeneous Poisson process. Poisson processes describe rare events [50], therefore, they are a suitable framework to describe mutations. We use the Poisson process to determine the time points of the mutations. At the respective time points, one cell acquires a new mutation and gives rise to a new clone. This founder cell is chosen from the present clones according to their contribution *α*_*i*_ to the total rate *α*. Self-renewal and proliferation rates of the new clone are chosen based on the parameters of the founder cell. We assume that the traits (self-renewal and proliferation rates) of the new clone are normally distributed with a predefined variance and the mean value corresponding to the parameters of the founder cell. Since biological parameters are restricted to a predefined interval, we use truncated normal distributions. A suitable interval for proliferation rates is between one division per year and one division per day [46] and the fraction of self-renewal is by definition between zero and one.

At the time of its birth a new clone consists of one mitotic and zero post-mitotic cells. Due to the stochasticity of cell fate decisions or due to cell death it is possible that the new clone becomes extinct. For example, if the newly generated mitotic cell divides and gives rise to two differentiated progeny, the new clone will eventually extinct, since there exist no more mitotic cells. We use the theory of Galton-Watson processes to calculate the probability of extinction of new clones. We adopt the methodology from ref. [51], which is similar to the approach used in ref. [31]. We notice that a clone eventually becomes extinct if it has no mitotic cells. If a mitotic cell divides, with the probability *a* ^2^ both progeny are mitotic cells, with the probability 2(1−*a*)*a* one progeny is a mitotic cell and with probability (1−*a*)^2^ both progeny are fully differentiated. By *a* we denote the fraction of self-renewal of the mitotic cells. The probability generating function for the number of mitotic progeny is *f*(*x*)=*a* ^2^ *x* ^2^+2*a*(1−*a*)*x*+(1−*a*)^2^. If we assume in addition that the parent cell dies with a probability *d* during division the probability generating function is *f*(*x*)=(1−*d*)(*a* ^2^ *x* ^2^+2*a*(1−*a*)*x*+(1−*a*)^2^)+*d*.

If we assume that cells of the new clone divide at discrete times *iT*, $i\in \mathbbm {N}$, where *T* is the average generation time, we can use the theory of Galton-Watson processes to calculate the extinction probability. We know that the extinction probability is the smaller solution of *f*(*x*)=*x* [28]. We neglect cell death and obtain for the extinction probability

$${} p_{e}(a)=\frac{2a^{2}-2a+1}{2a^{2}}-\sqrt{\left(\frac{2a^{2}-2a+1}{2a^{2}}\right)^{2}-\frac{(1-a)^{2}}{a^{2}}}. $$

We notice that *p*_*e*_<1 if *a*>0.5. For each new clone we calculate the extinction probability based on its self-renewal rate at the time of emergence. With probability 1−*p*_*e*_ the new clone is introduced to the system by adding two equations to the system describing dynamics of mitotic and post-mitotic cells of the new clone. With probability *p*_*e*_ the new clone is not introduced to the system since it becomes extinct.

According to biological data suggesting that all leukemic cells are derived from one leukemic or preleukemic clone [12], we neglect mutations in the healthy cells. Instead we introduce one leukemic founder clone at *t*=0 to the system and study the dynamics of the founder clone and the clones arising from it. Parameters of the founder clone are assumed to be normally distributed with the means equal to parameters of healthy hematopoietic cells.

### Parametrization

The parameters of the hematopoietic system are adopted from ref. [22]. In the following we shortly describe the calibration. For details see ref. [22] and the references therein. The numbers of myeloid mitotic ($\bar {c}_{1}$) and post-mitotic ($\bar {c}_{2}$) cells and the neutrophil clearance (*d*_2_) are taken from literature. Analytical expressions of the steady state cell counts allow to calculate *p*_*c*_ based on $\bar {c}_{1}$, $\bar {c}_{2}$ and *d*_2_. The parameter *k* can be expressed as a function of known quantities and $\hat {a}_{c}$. To obtain an appropriate value for $\hat {a}_{c}$, we simulate stem cell transplantation. We know that after transplantation of a dose of 3 to 5·10^6^ cells per kg body weight, patients recover to 5·10^8^ neutrophils per liter of blood within 15 days. To set the initial condition we assume that the ratio of myeloid to erythroid cells in the transplant is as in the healthy marrow between 2:1 and 4:1. We choose *a*_1_ such that we observe recovery after 2-3 weeks. This results in the following parameters: ${\hat {a}_{c}}=0.87$, *p*_*c*_=0.45/*d* *a* *y*, *d*_*c*_=2.3/*d* *a* *y*, *k*=1.85·10^−9^, *d*_*l*_=0.5/*d* *a* *y*. These parameters refer to healthy cells. Parameters of the leukemic cells are chosen randomly according to normal distributions.

For the simulations the rate *ν* is varied between 5·10^−8^ and 10^−11^. Standard deviations for the normal distributions are varied between 0.001 and 0.1. The standard deviations and mutation rates used to obtain the figures are specified in the figure captions. Stopping criteria for simulations are either decline of healthy blood cells to less than 5 % of the healthy steady state value or a simulated time span that exceeds 100 years of age for a given patient. An example simulation is depicted in Fig. 2 b.

## Results

Simulations over wide parameter ranges, including variation of mutation rates over several orders of magnitude, show that the phenomena presented below are robust with respect to the parameter choice.

### Self-renewal rate of significant clones increases during the course of the disease

**Fig. 3:**
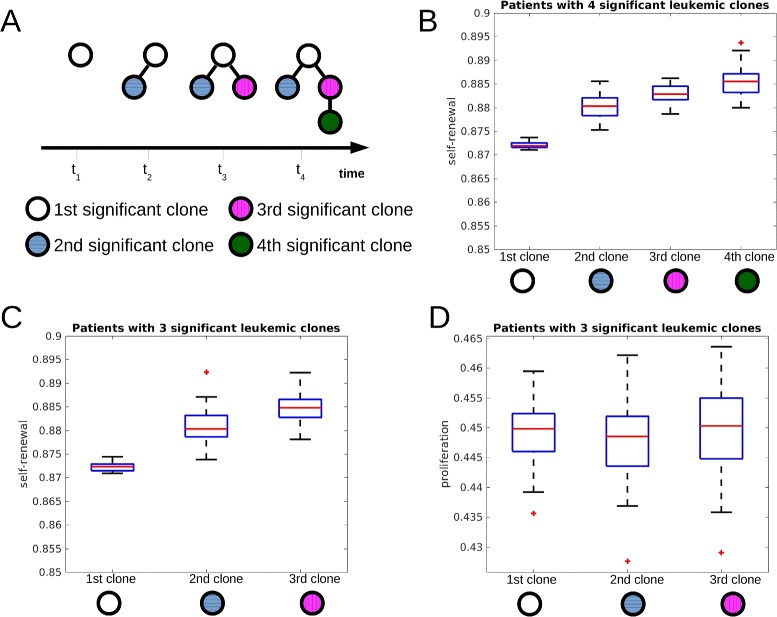
Self-renewal rate of significant clones increases during the course of the disease. The figure is based on 600 simulated patients. **a** Time evolution of one simulated patient who developed 4 clones during the course of the disease. The first, second, third and fourth significant clone are depicted using different colors. **b** Self-renewal rates of the first, second, third and fourth significant leukemic clone of the subgroup of patients harboring 4 significant clones at the end of the simulation (reduction of healthy cell count by 95 %). The self-renewal rates differ significantly (*p*<0.05 in t-test) between first and second, second and third, third and fourth clone. **c** Self-renewal rates of the first, second and third significant leukemic clone of the subgroup of patients harboring 3 clones at the end of simulations. The self-renewal rates differ significantly between the clones (*p*<0.05 in t-test). **d** Proliferation rates of the first, second and third significant leukemic clone of the subgroup of patients harboring 3 clones at the end of simulation. The proliferation rates do not differ significantly between the clones (*p*>0.05 in t-test). Parameters: mutation rate *ν*=5·10^−9^, self-renewal and proliferation rates of the new clones are normally distributed with the means of the distributions equal to proliferation and self-renewal rates of the parent clone and standard deviation equal to 0.005. The central mark is the median, the edges of the box are the 25th and 75th percentiles, points are drawn as outliers if they are larger than *q*_3_+1.5(*q*_3_−*q*_1_) or smaller than *q*_1_−1.5(*q*_3_−*q*_1_), where *q*_1_ and *q*_3_ are the 25th and 75th percentiles, respectively

We first ask how cell properties evolve during the course of the disease. For this purpose, we compare self-renewal and proliferation rates of the significant clones of 600 simulated patients. The significant clones emerge at different time points due to mutations. We count the significant clones in the order of the time of their emergence. Simulation results imply that in more than 95 *%* of patients the self-renewal rate of the second emerging significant clone is larger than the self-renewal rate of the first emerging significant clone. The self-renewal rate of the third significant clone is larger than the self-renewal rate of the second significant clone and so forth. Interestingly, proliferation rates do not differ significantly among significant clones. This finding confirms that high self-renewal rate is beneficial for expansion of clones. Previous simulation studies have shown that high self-renewal rate might be related to poor prognosis and high resistance to therapy [22, 23]. Simulation results imply that clones become more aggressive during the course of the disease. In the remaining 5 *%* of simulated patients, the following phenomena have been observed: (i) in very rare cases (less than 1 *%*) a slight reduction in the self-renewal rate is compensated by an increase in proliferation rate, (ii) two new clones emerge within a short time span, the self-renewal rate of both clones is larger than the self-renewal rate of the parent clones, but one of the emerging clones has a slightly reduced self-renewal rate compared to the other. In this case both new clones have growth advantage at the time of their origin and, therefore, grow to a significant size. The increasing self-renewal of clones over time is the result of a selection process. The selection was studied numerically in [22] and proved in [52] for a multi-clonal system in absence of mutations. When a new clone arises due to a mutation, its self-renewal and proliferation rates can be larger or smaller than the corresponding rates of the parent clone. They are chosen according to normal distributions with constant standard deviation and means equal to the parameter values of the parent clone. It is not straightforward to predict whether progeny clones with higher self-renewal, higher proliferation or both have more competitive advantages. Simulation results show that among the newly arising clones with random proliferation and self-renewal values those with high self-renewal are more competitive than those with high proliferation. The results are depicted in Fig. 3.

### Properties of the first clone determine if there is no outbreak of the disease, a monoclonal disease, or clonal diversity

**Fig. 4:**
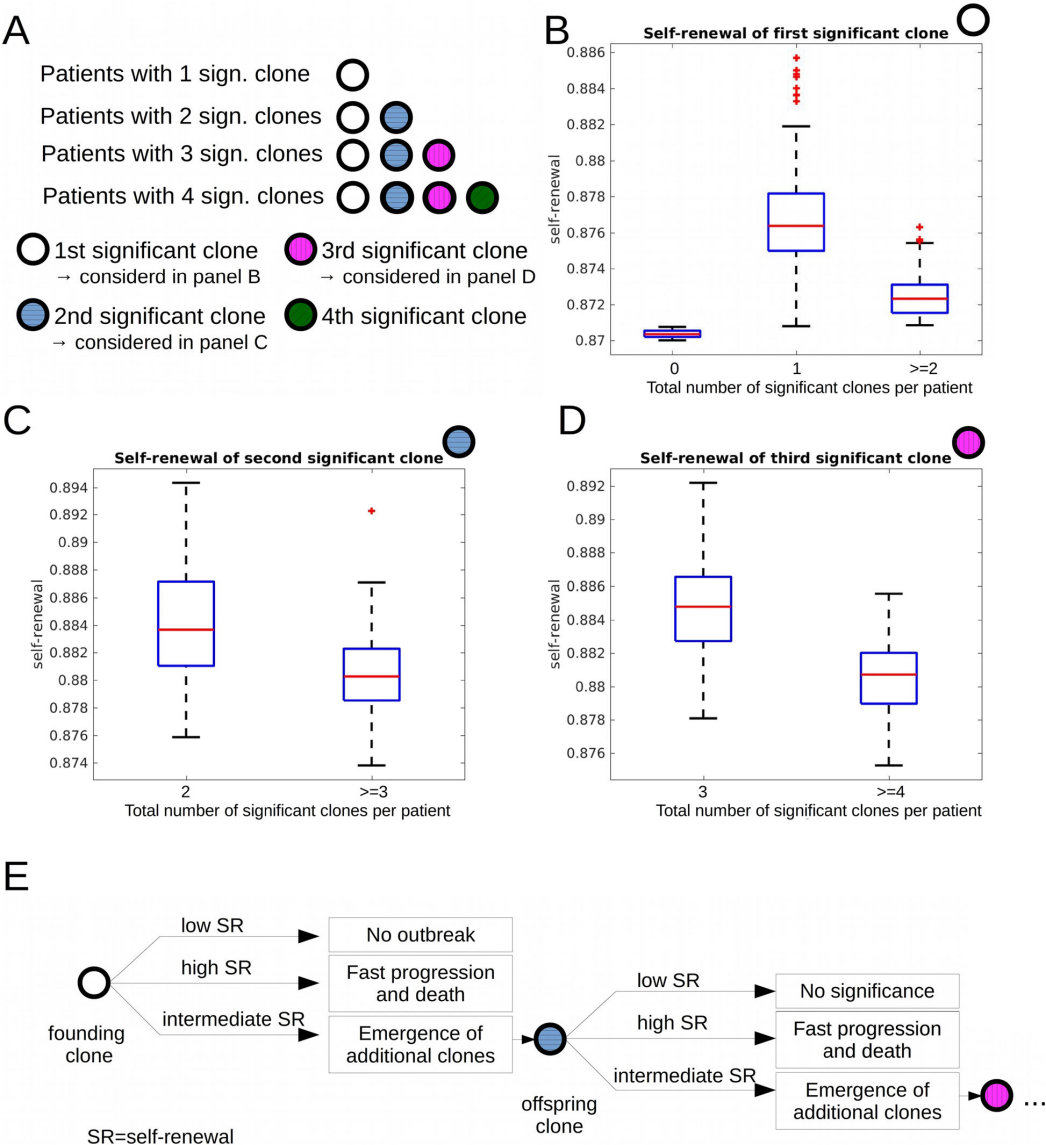
Impact of self-renewal rate on the total number of significant clones. Data from 600 simulated patients. **a** Overview over panels (**b**)–(**d**). **b** We compare self-renewal rate of the first significant clone in two patient groups. Group 1: patients harboring only one significant clone throughout the disease. Group 2: patients harboring more than one significant clone. The self-renewal rate of the first significant clone is significantly higher in group 1. Leftmost plot in (**b**): If the self-renewal rate of leukemic clones is close to the self-renewal rate of healthy cells, no significant clones emerge. **c** We compare the self-renewal rate of the second significant clone in two patient groups. Group 1: patients harboring two significant clones throughout the disease. Group 2: patients harboring more than two significant clones. The self-renewal rate of the second significant clone is significantly higher in group 1. **d** We compare the self-renewal rates of the third emerging significant clone in two patient groups. Group 1: patients harboring three significant clones throughout the disease. Group 2: patients harboring more than three significant clones. The self-renewal rate of the third significant clone is significantly higher in group 1. **e** Explanation of data in (**b**)–(**d**): Clones with high self-renewal rates lead to fast progression of the disease and death before new significant clones can emerge. Clones with small self-renewal rates never grow to a significant size. Clones with intermediate self-renewal rates grow with moderate speed and their offspring have enough time to grow to a significant size. Proliferation rates do not differ significantly between all considered groups. Parameters: mutation rate *ν*=5·10^−9^, self-renewal and proliferation rates of the new clones are normally distributed with the mean of the distributions equal to proliferation and self-renewal rates of the parent clone and standard deviation equal to 0.005. Significance: *p*<0.05 in t-test

The number of significant clones varies among the patients [12]. We compared self-renewal and proliferation rates of the significant clones in simulated patients to investigate how these properties impact the total number of significant clones per patient. As mentioned above, we suppose that all leukemic clones originate from a single founder clone which itself is derived from healthy hematopoietic cells. Simulation results imply that the self-renewal rate of the founder clone has a major impact on the total number of significant clones emerging during the course of the disease. If the founder clone has a high self-renewal rate it has the ability to expand fast. This fast expansion leads to a clinical disease and potential death of the patient before additional significant clones can emerge. In case of very small self-renewal rate, the founder clone expands slowly and the disease does not become significant throughout the life span. In case of intermediate self-renewal rate of the founder clone, multiple significant clones arise. If the founder clone gives rise to a clone that grows to a significant size over time, the self-renewal rate of this second significant clone determines whether a third significant clone will arise. If the self-renewal rate of the second significant clone is high, fast expansion and progression of the disease follow. The remaining life time of the patient is too short for emergence of additional significant clones. If the self-renewal rate of the second significant clone has intermediate values, disease progression is slow and more significant clones can emerge. This principle is extended to a higher number of leukemic clones: If the self-renewal rate of the first *n* significant clones is intermediate, the probability is high that additional clones will emerge. If one clone among the first *n* significant clones has a high self-renewal rate, progression is fast and no more clones emerge. Clones with small self-renewal rate never grow to a significant size. Proliferation rates of the clones do not have a significant impact on the total number of clones. Simulations show that if we restrict self-renewal rate of the leukemic founder clone to intermediate values, e.g., between 0.872 and 0.875, the number of clones per patient increases. The results are summarized in Fig. 4.

### Self-renewal rate increases with an increasing depth of clones

In leukemia patients, clonal hierarchies show considerable interindividual variations [12]. We ask how the properties of clones influence the depth of the clonal hierarchy. We assume that the founder clone has depth 1. Clones that have acquired *k* additional mutations in comparison to the founder clone have depth 1+*k*. Depth of a clonal hierarchy is understood as the maximal depth of its clones. Simulations indicate that the self-renewal rate of the clones increases with their depths. This is plausible: To be able to give rise to new clones, a given clone has to achieve a critical mass of cells. Only then it is probable that cells of this clone mutate and give rise to offspring clones. To achieve the critical mass, a clone needs properties favorable for efficient expansion. This occurs if and only if its self-renewal rate is increased compared to its mother clone, since otherwise the mother clone outgrows its offspring.

Simulations show that a deep clonal hierarchy requires a step-wise increase of self-renewal rate with each mutation. The step size determines how deep the hierarchy will be. If the step size is too small, offspring clones grow slowly and it takes long time until they and their offspring grow to a significant size. In this case the parent clone remains dominant in size and is responsible for disease progression. If the step size is large, the offspring clones grow fast and the patient dies before potential new offspring achieve a significant size. In both cases the hierarchy is flat. Only if self-renewal rate increases by steps of intermediate size, deep hierarchies are observed. In this case, the offspring clones have a sufficient growth advantage compared to their parents but they grow slow enough for their offspring to achieve a significant size and to give rise to new clones.

Self-renewal rate of the significant clone which appears first has a major impact on the depth of the hierarchy. If it has a high self-renewal rate, the disease progresses fast and the patient dies before offspring achieve a critical mass. In case of small self-renewal rate of the first clone, deep hierarchies can emerge, supposed that it gives rise to offspring with higher self-renewal rates. Analogously the properties of a clone of depth 2 determine whether a clone of depth 3 can emerge. The proliferation rate has no impact on the depth of the hierarchy.

**Fig. 5:**
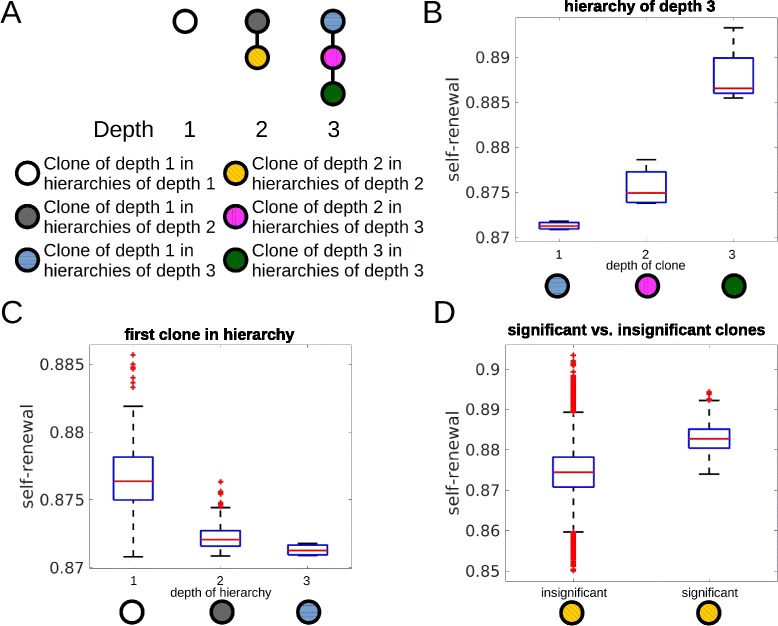
Impact of the self-renewal rate on the depth of the hierarchy. The figure is based on 600 simulated patients. **a** Examples for hierarchies of different depths. Colors are used to visualize clones of different depths. **b** Self-renewal rate of significant clones increases significantly (*p*<0.05 in t-test) with the depth of the clones in hierarchies. Considered are only patients with clonal hierarchies of depth 3. **c** Properties of the first clone in the hierarchy decides about the depth of the hierarchy. Only if self-renewal rate of the first clone is small enough deep hierarchies emerge. If self-renewal rate of the first clone is high, disease progression and death occur before deep hierarchies can establish. **d** Comparison of self-renewal rates of significant and insignificant clones of depth 2. Self-renewal rates of insignificant clones is significantly smaller than self-renewal rates of significant clones. This demonstrates that clones do not become significant if their self-renewal rate is too small. Some of the insignificant clones show high self-renewal rates. These clones have originated late during the disease and could not grow to a significant size before death of the patients. If proliferation rate is very slow, clones with high self-renewal cannot grow to a significant size. Proliferation rate has no impact on the depth of the hierarchy. Parameters: mutation rate *ν*=5·10^−9^, self-renewal and proliferation rates of the new clones are normally distributed with the mean of the distributions equal to proliferation and self-renewal rates of the parent clone and standard deviation equal to 0.005

High self-renewal rate is potentially linked to poor prognosis and fast progression of the disease [22, 23]. If we consider the maximum of self-renewal capacity over all significant clones, the simulations imply that it increases significantly with the depth of the clonal hierarchy. Therefore, our study suggests that the depth of the clonal hierarchy could be considered a prognostic parameter. Since in our simulations deep hierarchies are linked to high self-renewal rates, our results suggest that deep hierarchies could be linked to poor prognosis. Interestingly, there is no correlation between the total number of significant clones and the maximal self-renewal rates of the significant clones. The results are summarized in Fig. 5.

### Cooperativity of mutations might explain emergence of deep hierarchies

In the patients investigated in ref. [12], hierarchies of depths between 3 and 5 have been detected. Due to the findings described in the previous paragraph, emergence of such hierarchies requires a coordinated increase of the self-renewal rate with each acquired mutation. Appearance of clones with too high self-renewal rates leads to fast progression and death before deep hierarchies can be established. Therefore, the existence of deep hierarchies is not compatible with mutations that lead to large changes of cell properties. Indeed, if we assume that traits of mutated clones are uniformly distributed in the trait space, deep hierarchies are never observed in simulations.

This observation raises the question which probability distributions are suitable choices to model the effect of mutation in the trait space. We have investigated the assumption that the traits of the new clone follow normal distributions with means equal to the traits of the cell that gave rise to the new clone. Depending on the assumed standard deviations of the normal distributions we can observe hierarchies of varying depths. If the standard deviations are too large, the hierarchies remain flat, since aggressive clones that lead to fast progression and death emerge early in the disease. If the standard deviations are too small, the traits of the offspring clones are very close to the traits of the parent clones. Therefore, the offspring clones have little growth advantage compared to their parent clones and consequently need long time to grow to a significant size and to produce offspring growing to a significant size. In these cases the hierarchy of significant clones remains flat. Only if the standard deviation is within a limited range, a considerable number of patients with deep hierarchies is observed in the simulations. In acute leukemias, where genetic instability is rare, generation of large numbers of mutated cells and selection of those which exactly fit the properties required to establish deep hierarchies is not a realistic scenario, since mutation rates are relatively low, compared to other cancers. Leukemias show high interindividual genetic variability. The assumption that all described mutations will lead to exactly those changes in the self-renewal rates that are required to establish deep hierarchies seems also improbable.

If we assume that the standard deviation of the normal distribution according to which the traits of the offspring are chosen increases with each mutation, deep hierarchies become a more frequent event. This is plausible since small standard deviations for the first mutation avoid emergence of clones that show fast expansion and subsequent death of the patient. Step-wise increase of standard deviation with each mutation allows the offspring to gain sufficient growth advantage compared to the parent clones that they can grow to a significant size. At the same time large jumps leading to aggressive clones remain rare.

The assumption that jump sizes in the trait space increase with the number of mutated genes in a cell seems plausible from a biological point of view. Cells are known to have redundant pathways for regulation of important functions. Perturbation of one pathway by a mutation might therefore lead to only small jumps in the trait space, whereas subsequent perturbation of multiple pathways may lead to larger jumps in the trait space. This means that the presence of a mutation facilitates the occurrence of large effects due to an additional mutation. In this sense the different mutations are cooperative.

**Fig. 6:**
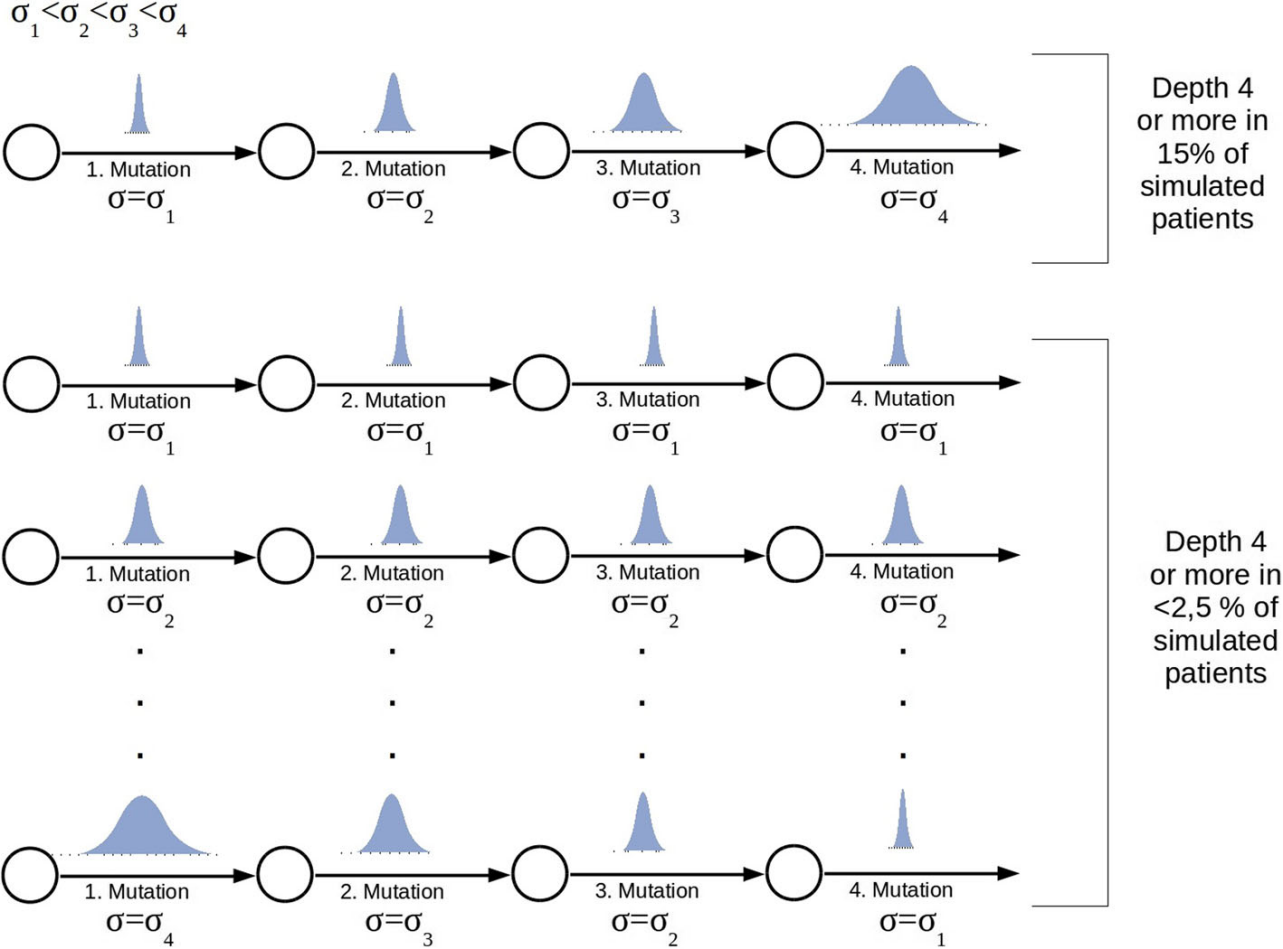
Impact of cooperativity between mutations on depth of the hierarchy. The figure is based on 100 simulated patients. The number of patients harboring a clonal hierarchy of depth 4 or more is maximized, if the jumps in the trait spaces increase with the number of mutations. Parameters: Self-renewal and proliferation rates of the leukemic founder clone are drawn from normal distributions with mean values equal to proliferation and self-renewal rates of healthy cells and standard deviation *σ*_1_=0.0014. First mutation: self-renewal and proliferation rates of the new clone are normally distributed with the means of the distributions equal to proliferation and self-renewal rates of the founder clone and standard deviation *σ*_2_=5·*σ*_1_. Second mutation: self-renewal and proliferation rates of the new clone are normally distributed with the means of the distributions equal to proliferation and self-renewal rates of the parent clone and standard deviation *σ*_3_=20·*σ*_1_. Third and higher mutations: self-renewal and proliferation rates of the new clone are normally distributed with the means of the distributions equal to proliferation and self-renewal rates of the parent clone and standard deviation *σ*_3_=100·*σ*_1_. Mutation rate *ν*=5·10^−9^

The importance of cooperativity is underlined by the following simulation experiment: We assume that the probability of large jumps in the trait space increases with the number of accumulated mutations. We model these effect using normal distributions with increasing standard deviations *σ*_1_<*σ*_2_<*σ*_3_…, i.e., the size of the jump in the trait space due to the first mutation is given by a normal distribution with standard deviation *σ*_1_, the jump due to the second mutation by a normal distribution with standard deviation *σ*_2_ etc. We simulate the emergence of clonal hierarchies under these assumptions. We repeat simulations under modified assumptions, for example, we assume that for all mutations the size of the jump in the trait space is given by a normal distribution with standard deviation equal to *σ*_1_ or equal to *σ*_2_ etc. We run simulations for all possible permutations of *σ*_1_,*σ*_2_,*σ*_3_…. Comparison of simulation results shows that the number of patients harboring hierarchies of depth 4 or more is maximized if standard deviations increase from one mutation to another. The results are depicted in Fig. 6.

### Impact of the mutation rates and probability distributions on the clonal hierarchies

We studied the architecture of clonal hierarchies for several mutation rates. For increased mutation rates the total number of clones increases. Interestingly, the number of significant clones increases only moderately if the mutation rates are varied over several orders of magnitude; for example, if the rate increases from 5·10^−10^ to 5·10^−9^, the mean number of all clones increases by a factor of 8, whereas the mean number of significant clones increases only by 1. In all cases the number of significant clones was smaller than 15 and for 80 % of the patients smaller than 10. This is in line with the observation of clone numbers in experimental studies [11, 12]. This finding underlines the role of competition between the different clones. The competition selects among an increasing total number of clones always a small number of significant clones.

Simulation results imply that patients with less aggressive clones and without disease outbreak are over-represented in case of small mutation rates. Patients with highly aggressive clones and fast disease progression are over-represented in case of high mutation rates. This is plausible: The higher the mutation rate, the more clones are generated per unit of time. The probability that at least one clone per patient has favorable growth properties increases with the number of generated clones. Similarly the probability that highly aggressive clones and fast disease progression occur increases with increasing mutation rate. For all mutation rates we observed that clonal hierarchies are flat in case of fast disease progression and in case of very slow disease progression compared to cases with intermediate disease progression.

Increased mutation rates act in favor of deep hierarchies. Nevertheless this effect is mild and the mean depth increases by 1 if the mutation rate increases by a factor of 10. This observation can be explained by the fact that high mutation rates lead to increased numbers of leukemic clones. Therefore, the probability that a clone gives rise to at least one offspring with favorable growth properties increases. As discussed above, probability distributions according to which the traits of new clones are determined have an important effect on the depth of the hierarchy. If uniform distributions over the possible parameter range are chosen, deep hierarchies are very rarely observed. Also the total number of significant clones is decreased. Similarly, if standard deviations of normal distributions increase over a certain threshold, the mean number of significant clones slightly decreases, e.g., the number of significant clones decreases by 1 if the standard deviations are increased from 0.01 to 0.05.

### Comparison to data

**Fig. 7:**
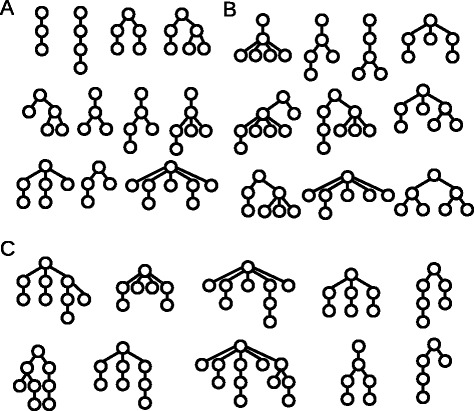
Examples for the clonal architecture detected in leukemic patients and simulations. Each tree corresponds to one patient. **a** Clonal hierarchies detected in patients from [12] and in simulations. **b** Clonal hierarchies detected in simulations but not in patients from [12]. **c** Clonal hierarchies detected in patients from [12] but not in simulations. The reconstruction of the clonal architecture from genetic measurements is not always unique. In case of ambiguity only one possibility is shown in the figure. Parameters were set as specified in the caption of Fig. 6

We compare the structure of the clonal hierarchy obtained by simulations of our model with the clonal hierarchies in 30 patients from [12]. The patients’ data are based on genetic studies. To take into account the limitations of the experimental methods, we compare the data only to significant clones observed in the numerical simulations. For more than 60 *%* of the patients the clonal hierarchies are reproduced by our model. Besides, we observe both hierarchies obtained in numerical simulations that are not found in the patients’ data and hierarchies in the data which could not be reproduced numerically. The latter could be explained by dynamic variation over the hierarchies in time. The hierarchy at diagnosis only reflects the situation at one time point. In simulation results we only considered the hierarchies at three time points per patient, namely at the time points when mature cell counts have decreased by 5, 50 and 95 *%*. In approximately 30 *%* of the patients with hierarchies not reproduced by the simulations, patient data could be reproduced if one clone existing in the simulations with an insignificant size would grow to a significant size. Examples are provided in Fig. 7.

## Discussion

We propose a mathematical model to study the emergence of clonal heterogeneity in acute leukemias. The model considers the interactions of multiple leukemic clones with healthy hematopoiesis and the emergence of new clones due to mutations. We use computer simulations to study the impact of leukemic cell self-renewal and proliferation rates on the structure of the clonal hierarchy. At the same time, the model provides insights into how the properties of clones at different positions in the clonal hierarchy differ. These questions are clinically relevant, since the patients’ prognosis and the treatment response may depend on the properties of the leukemic cells [23].

Model simulations suggest that the self-renewal rate of leukemic clones has a major impact on the structure of the clonal hierarchy, whereas proliferation rates show no significant influence. The self-renewal rate of the emerging clones increases during the course of the disease. There is evidence that a high self-renewal rate of clones may be linked to poor prognosis [23]. In this sense, clones appearing later during the disease are more aggressive than those present at the beginning of the disease. Similarly, simulations suggest that the self-renewal rates of clones increase with increasing depth of the hierarchy, whereas proliferation rates do not depend significantly on the depth of clones in the hierarchy. Simulations of large patient groups suggest that there might exist a significant relationship between the depth of the clonal hierarchy and the maximal self-renewal rate. This finding suggests to evaluate the depth of the clonal hierarchy as a potential marker for patient prognosis.

Mutations detected in acute leukemias act at different regulatory levels. There is evidence that many of them lead to increased self-renewal. Important examples for genes where mutations lead to increased self-renewal are the chromatin modifiers *TET2* [53], *DNMT3A* [54] and *MLL* [55] or the transcription factors *C/EBP* *α* [56], *RUNX1/CBF* *β* [57, 58] and factors encoded by the *HOX* genes, e.g., as *NUP98-HOXA9* [59]. Other examples include the isocitrate dehydrogenase *IDH1* [60], the *NRAS* gene [61] or the multi-functional protein *NPM1* [62]. Importantly, more than one of these mutations can occur in the same cell [63, 64]. This is in line with the step-wise increase in self-renewal observed in the model simulations.

Emergence of the clonal hierarchy is a dynamic process. Model simulations show that the properties of the existing clones have an impact on the structure of the clonal hierarchy in the future. Presence of aggressive clones with high self-renewal rates leads to fast progression of the disease. The short remaining lifespan of the patient limits the number of new clones that can emerge and grow to a significant size. Therefore in presence of aggressive clones, the clonal hierarchies consist of a relatively small number of clones. On the other hand, if the self-renewal rates of new clones is very close to the self-renewal rate of the parent clone, the new clone expands slowly and takes a long time to reach a significant size. Therefore, mutations causing only small changes in self-renewal rates also lead to small numbers of significant clones and flat hierarchies.

The model simulations suggest that the emergence of deep clonal hierarchies is a complex process. To give rise to offspring, a clone requires a critical mass, otherwise it is unlikely that a clone acquires new mutations. A deep hierarchy is created if new clones have high enough self-renewal rates to grow to a critical mass before the patient dies, but not too high self-renewal rates to avoid fast progression and death before the new clones can produce their own offspring. Simulations imply that these constraints are rarely met if one assumes that different mutations act independently, i.e., the properties of a new clone compared to the parent clone follow the same random distribution for each mutation. This limitation can be overcome if cooperativity of mutations is assumed, i.e., if the probability that a new mutation leads to large changes of cell properties increases with the number of mutations that the cell has experienced in the past. Since deep hierarchies are frequently observed in patients (ref. [12]) it seems very plausible that cooperativity of mutations plays a role during evolution of the acute leukemias. In the presence of cooperativity we observe a significant increase in the emergence of deep hierarchies and also in the interindividual heterogeneity of clonal hierarchies. Assuming cooperativity allows to reproduce many of the clonal hierarchies detected in patients from ref. [12] by model simulations.

Computer simulations of the proposed model suggest that increasing mutation rates have only a limited impact on the number of significant clones. Although the total number of clones increases with increasing mutation rates, the number of significant clones remains approximately constant. This observation can be explained by the strong competition among leukemic clones. This is in line with experimental studies [11, 12] which report similar numbers of clones in different patients.

Genetic and epigenetic mechanisms are closely intertwined in leukemogenesis. Genes involved in epigenetic encoding are often mutated in leukemias [65, 66]. In addition, epigenetic changes can be driven by mutations which are not directly linked to the epigenetic machinery such as IDH1/2 or several transcription factors [67]. In principle, epigenetic mechanisms can be included in the proposed modeling framework. It has to be clarified whether epigenetic effects can be modeled similar to mutations as random, independent, discrete jumps in the trait space. This may be true for epigenetic changes that mimic genetic mutations such as in the case of *DNMT3A* [68]. In other cases it might be more appropriate to model epigenetic modifications as drift or diffusion in a continuous trait space as done in [33]. Plasticity and potential reversibility are important hallmarks of epigenetic changes. The possibility that clones readopt the traits of their ancestors can be included easily into the model. The same holds for the potentially different time scales of genetic and epigenetic modifications.

Mathematical modeling of clonal hierarchies can be a helpful tool, since it allows to link cell properties to a position in the clonal hierarchy. This may have a clinical relevance. Model simulations imply that the clonal hierarchy allows drawing conclusions about the course of the disease, even if the latter is known only at one time point. This work has the following clinical implications which could be discussed and evaluated in clinical trials: (i) A small number of clones detected at diagnosis may argue in favor of a rapidly progressing disease with aggressive clones. These patients may have poor prognosis and potentially a rapid progression after standard treatment or a poor treatment response. (ii) Deep hierarchies e.g., of order 5 or more, may argue for a long course of the disease before diagnosis. The probability for the presence of aggressive clones is high, which may limit prognosis. Nevertheless clones at the top of the hierarchy may respond to the treatment. (iii) Hierarchies of intermediate depth, e.g., 3 or 4 may argue in favor of long courses and limited aggressiveness of the clones. Patients with good prognosis may be over-represented in this group.

## Conclusion

Emergence of heterogeneity in acute leukemias is a complex process. Multi-clonality and deep hierarchies emerge only from leukemic clones with intermediate self-renewal. Emergence of deep hierarchies is facilitated by a cooperativity of different mutations. There is no correlation between the position of a clone within the hierarchy and its proliferation rate. Self-renewal rates of leukemic clones increase with the depth of mutation hierarchy. Therefore, the structure of the hierarchy may serve as a marker for patient prognosis.

## Reviewers’ comments

### Reviewer’s report 1: Marek Kimmel, Rice University, Houston, TX, USA

#### Reviewer summary:

The paper is focused on modeling the clonal structure of driver mutations in leukemias. It is based on a previous model by the same group, which is augmented by new mutations appearing at random. The topology of the resulting clonal pedigrees is compared to literature data, notably ref. [12]. The results are plausible in that the collection of structures generated by the model matches that in ref. [12]. I enjoyed reading the paper, which is certainly sufficiently novel to be published.

#### Reviewer recommendations to authors:

1. The main difficulty I see at the current stage of development of the model is that the simulated mutations are not identified with any particular “real” drivers. At least some comments on the subject will be helpful.

Authors’ Response: *We provide an overview of leukemic (driver) mutations that lead to increased self-renewal rates. Serial acquisition of such mutations could explain the step-wise increase in self-renewal rates observed in the model. We added the following paragraph to the discussion (p. 14, lines 23–34): Mutations detected in acute leukemias act at different regulatory levels. There is evidence that many of them lead to increased self-renewal. Important examples for genes where mutations lead to increased self-renewal are the chromatin modifiers TET2* [53], *DNMT3A* [54] *and MLL* [55] *or the transcription factors* *C/EBP* *α* [56], *RUNX1/CBF* *β* [57, 58] *and factors encoded by the HOX genes, e.g., as NUP98-HOXA9* [59]. *Other examples include the isocitrate dehydrogenase IDH1* [60], *the NRAS gene* [61] *or the multi-functional protein NPM1* [62]. *Importantly, more than one of these mutations can occur in the same cell* [63, 64]. *This is in line with the step-wise increase in self-renewal observed in the model simulations.*

2. The authors identified a plausible mechanism of selection of clones by assuming that faster clones are not seen in the patient population, since they lead to a sooner death, while on the other hand the slowest clones do not show since the patient may die of competing risks before he/she is diagnosed. It would be nice to see the sensitivity of this mechanism to changes in clone mutation rate distribution, for example.

Authors’ Response: *We run simulations for different mutation rates. The simulations imply that this mechanism is preserved. We added a paragraph to the* Results *section (p. 12 line 53 - p. 13 line 9): Simulation results imply that patients with less aggressive clones and without disease outbreak are over-represented in case of small mutation rates. Patients with highly aggressive clones and fast disease progression are over-represented in case of high mutation rates. This is plausible: The higher the mutation rate, the more clones are generated per unit of time. The probability that at least one clone per patient has favorable growth properties increases with the number of generated clones. Similarly the probability that highly aggressive clones and fast disease progression occur increases with increasing mutation rate. For all mutation rates we observed that clonal hierarchies are flat in case of fast disease progression and in case of very slow disease progression compared to cases with intermediate disease progression.*

3. I understand that once a mutation (= driver mutation?) arises, the clone it initiates survives. This is of course true only of some of the clones. The problem can be fixed by adjusting the rate of the Poisson process by the probability of non-extinction of the clone, as it was done for example in the models in Bozic et al. (2010) or Kimmel and Corey (2013). It would be interesting to know how such adjustment might change the effective mutation rates.

Authors’ Response: *We have adopted the approach described in Kimmel and Corey (2013) to take into account extinction probabilities. The introduction of extinction probabilities leaves our main results (increase of self-renewal over time, increase of self-renewal with increasing depth of the hierarchy) unchanged. Effective mutation rates decrease if the extinction of clones is modeled. This leads to a later disease outbreak. The frequency of deep hierarchies is reduced in the model with extinction probabilities. This can be explained by the fact that the extinction probability is higher if self-renewal of the new clone is small. Therefore clones with high self-renewal probability which lead to a fast course of the disease and patient death are favored. Corresponding explanations have been added to the* Methods *section on page 7 (lines 25–63).*

### Reviewer’s report 2: Tommaso Lorenzi, University of St Andrews, Scotland, UK

#### Reviewer summary:

Stiehl et al. present an in silico study of possible mechanisms which underpin the emergence and evolution of clonal heterogeneity in acute leukaemia. In line with previous work by the same authors, this study relies on a mathematical model consisting of a system of ordinary differential equations describing the evolution of hematopoietic cells and leukemic clones. From the modelling point of view, the main novelty lies in the fact that the authors incorporate here the effects of mutations occurring during genome replication. The manuscript is well organised and the results are presented in a clear and well-structured way. The work is based on computer simulations alone as the structure of the model would make it very hard to perform any qualitative analyses. The numerical results obtained are interesting and potentially relevant for the cancer research community. In particular, the comparison of numerical results with clinical data presented by Anderson et al. [Nature 469, 356-361, 2011] shades light on possible connections between clonal heterogeneity and the disease progression of leukaemia.

#### Reviewer recommendations to authors:

1. I would recommend that the authors discuss in more detail the originality of their model compared with the models presented in ref. [Werner et al., J. R. Soc. Interface 10, 20130349, 2013] and in the appendix of ref. [Stiehl et al., J. Royal Society Interface 11, 20140079, 2014].

Authors’ Response: *The model presented in Werner studies dynamics of mutations in non-stem cells. It is assumed that cell properties are identical for all cells carrying the same number of mutations. Most results of the work refer to neutral mutations that do not change the phenotype of the cells. The work neglects competition between the different mutated cells and feedback mechanisms regulating the number of healthy cells. The model proposed in our work is a more elaborated version of the model proposed in the Appendix of [Stiehl et al., J. Royal Society Interface 11, 20140079, 2014]. The implementation of our previous model did not allow to keep track of the clonal hierarchies. The assumption that traits of the new clones follow normal distributions may be more realistic than the uniform distributions used in our previous models. An additional new feature of the model presented in this manuscript is consideration of stochastic extinction of new clones. We added the following sentences to the* “Methods” *section:*

- *Page 3, lines 61–63: The main novelty lies in considering a time dependent number of leukemic clones and in tracking the structure of the clonal hierarchies.*
- *Page 4, lines 8–15: To model stochastic extinction of clones with favorable properties, we take into account their extinction probabilities using the theory of branching processes. Compared to the work presented in* [40], *which focuses on neutral mutations in non-stem cells without feedback regulation or competition, we are interested in the evolution of non-neutral stem cell mutations under competitive pressure of a nonlinear feedback mechanism.*

2. Although I agree that a careful description of the model parametrisation is presented in ref. [22] of the manuscript, I think it would be worthwhile to justify in more detail the choice of the parameter values in the subsection ‘Parametrization’.

Authors’ Response: *We added an additional paragraph to the section describing parametrization (p. 8, lines 15–33): In the following we shortly describe the calibration. For details see ref.* [22] *and the references therein. The numbers of myeloid mitotic* ($\bar c_{1}$) *and post-mitotic* ($\bar c_{2}$) *cells and the neutrophil clearance* (*d*_2_) *are taken from literature. Analytical expressions of the steady state cell counts allow to calculate* *p*_1_ *based on* $\bar c_{1}$, $\bar c_{2}$ *and* *d*_2_. *The parameter k can be expressed as a function of known quantities and* *a*_1_. *To obtain an appropriate value for* *a*_1_, *we simulate stem cell transplantation. We know that after transplantation of a dose of 3 to* 5·10^6^ *cells per kg body weight, patients recover to* 5·10^8^ *neutrophils per liter of blood within 15 days. To set the initial condition we assume that the ratio of myeloid to erythroid cells in the transplant is as in the healthy marrow between 2:1 and 4:1. We choose* *a*_1_ *such that we observe recovery after 2–3 weeks.*

3. I would suggest that Section 5 is removed with contents moved to Section 4; accordingly, I propose renaming Section 3 ‘Results and Discussion’ and Section 4 ‘Conclusions’.

Authors’ Response: *The titles of the sections are prescribed by the journal.*

4. I would recommend the authors indicate foreseeable extensions of the present work and future research perspectives. For instance, as recent experimental evidence suggests that epigenetic mechanisms can be implicated in the development of acute myeloid leukaemia [e.g., Jost et al., Leukemia 28, 1227-1234, 2014], the authors may want to discuss possible ways of extending their model to include the effects of epimutations.

Authors’ Response: *We added a paragraph to the Discussion (p. 15, lines 24–41): Genetic and epigenetic mechanisms are closely intertwined in leukemogenesis. Genes involved in epigenetic encoding are often mutated in leukemias* [65, 66]. *In addition, epigenetic changes can be driven by mutations which are not directly linked to the epigenetic machinery such as IDH1/2 or several transcription factors* [67]. *In principle, epigenetic mechanisms can be included in the proposed modeling framework. It has to be clarified whether epigenetic effects can be modeled similar to mutations as random, independent, discrete jumps in the trait space. This may be true for epigenetic changes that mimic genetic mutations such as in the case of DNMT3A* [68]. *In other cases it might be more appropriate to model epigenetic modifications as drift or diffusion in a continuous trait space as done in* [33]. *Plasticity and potential reversibility are important hallmarks of epigenetic changes. The possibility that clones readopt the traits of their ancestors can be included easily into the model. The same holds for the potentially different time scales of genetic and epigenetic modifications.*

### Reviewer’s report 3: Tomasz Lipniacki, Institute of Fundamental Technological Research, Polish Academy of Sciences,Warsaw, Poland

#### Reviewer summary:

Thomas Stiehl et al. propose a simple model of hematopoiesis of healthy and leukemic cell populations. The novelty is in inclusion of mutations that lead to time varying number of leukemic clones. The aim is to link the number of mutations that characterize a given clone with the self-renewal rate (fraction of divisions that do not lead to differentiation). Overall this is a nice study worth publishing, however some aspects require some clarification or more direct formulation.

#### Reviewer recommendations to authors:

1. The authors found that the self-renewal rate depend of the depth of clone in the hierarchy. It is not clear whether it is a simple consequence of assumption that clones pass self-renewal rate to the new clone emerging due to mutation. If so the clones with higher self-renewal rate that are more abundant have higher chance to give rise to new clones, and these clones will be characterized with higher self-renewal rate. The Authors write that traits of the new clone are normally or uniformly distributed (page 7), but no details are given.

Authors’ Response: *The increasing self-renewal of clones is the result of a selection process. We added an explanatory paragraph to the* Results *section (p. 9, lines 200–33): The increasing self-renewal of clones over time is the result of a selection process. The selection was studied numerically in* [22] *and proved in* [52] *for a multi-clonal system in absence of mutations. When a new clone arises due to a mutation, its self-renewal and proliferation rates can be larger or smaller than the corresponding rates of the parent clone. They are chosen according to normal distributions with constant standard deviation and means equal to the parameter values of the parent clone. It is not straightforward to predict whether progeny clones with higher self-renewal, higher proliferation or both have more competitive advantages. Simulation results show that among the newly arising clones with random proliferation and self-renewal values those with high self-renewal are more competitive than those with high proliferation. We have clarified the choice of parameters for the new clones in the* “Methods” *section:*

- *Page 7, lines 15–19: We assume that the traits (self-renewal and proliferation rates) of the new clone are normally distributed with a predefined variance and the mean value corresponding to the parameters of the founder cell.*
- *Page 8, lines 37–39: Standard deviations for the normal distributions are varied between 0.001 and 0.1. The standard deviations and mutation rates used to obtain the figures are specified in the figure captions.*

2. In “Parametrization” subsection Authors write that *a*_*c*_=0.87 (I think that authors mean $\hat {a}_{c}$ since *a*_*c*_ depends to the strength of the negative feedback i.e. number of cells), then they write that for Fig. 4 self-renewal is between 0.872 and 0.875?

Authors’ Response: *We corrected the notation. The maximal self-renewal of 0.87 refers to the self-renewal of healthy cells. We have clearly indicated this in the revised version. The range of self-renewal between 0.872 and 0.875 refers to self-renewal of leukemic cells. We added the following sentences:*

- *Page 8, lines 31–33:* ${\hat {a}_{c}}=0.87$, *p*_*c*_=0.45/*d* *a* *y*, *d*_*c*_=2.3/*d* *a* *y*, *k*=1.85·10^−9^, *d*_*l*_=0.5/*d* *a* *y*. *These parameters refer to healthy cells. Parameters of the leukemic cells are chosen randomly according to normal distributions*
- *Page 10, line 12: Simulations show that if we restrict self-renewal rate of the leukemic founder clone to intermediate values, e.g., between 0.872 and 0.875, the number of clones per patient increases.*

3. It is not clear how long are the simulations? When self-renewal rate multiplied by feedback strength s(t) is larger than 1/2, the cell sub-populations are growing. It is not clear whether the authors run the simulations long enough to reach the constant population size limit. A figure showing number of cells in each clone would be helpful.

Authors’ Response: *We added a panel showing an example for the time course of the leukemic clones to Fig. 2. As shown in* [22, 52], *the cell count in each clone converges asymptotically to an equilibrium, which is nonzero only in the case of clones with maximal fitness, i.e., the largest self-renewal. In clinical scenarios, patients die before the equilibrium is reached. Allowing mutations with increasing self-renewal not approaching one would lead to a dynamics that does not converge to an equilibrium. The latter is however an artificial example as far as biological applications are concerned.*

4. Since the clone starts from single founder cell, in the beginning of each clone ODE formalism is not adequate – some comment is needed here.

Authors’ Response: *Following the advice of Reviewer 1 (point 3.) we extended the model to take into account extinction probabilities of newly generated clones. For each new clone we calculate the extinction probability based on the theory of Galton-Watson Processes. The probability that a new clone is introduced to the system is equal to its probability of non-extinction.*

5. The conclusion in abstract that “clonal heterogeneity might impact the course of the disease” is very vogue.

Authors’ Response: *We have replaced this formulation by a more specific statement: Comparison of patient data and simulation results suggests that the self-renewal of leukemic clones increases with the emergence of clonal heterogeneity.*

6. Technical: Page 5 “Model equations for hematopoietic system” there is once *p*_*c*_ once *p* ^*c*^ – I think it denotes the same thing.

Authors’ Response: *Thank you. We corrected it.*

## Abbreviation

Resp.: Respectively
